# Sharing resources to advance translational research

**DOI:** 10.1242/dmm.049944

**Published:** 2022-10-31

**Authors:** Kirsty M. Hooper, Julija Hmeljak

**Affiliations:** The Company of Biologists, Bidder Building, Station Road, Histon, Cambridge CB24 9LF, UK

**Keywords:** Disease modelling, Human disease, Resource articles

## Abstract

The publication of Resource articles is essential for the dissemination of novel, or substantially enhanced, tools, techniques, disease models, datasets and resources. By sharing knowledge and resources in a globally accessible manner, we can support human disease research to accelerate the translation of fundamental discoveries to effective treatments or diagnostics for diverse patient populations. To promote and encourage excellence in Resource articles, Disease Models & Mechanisms (DMM) is launching a new ‘Outstanding Resource Paper Prize’. To celebrate this, we highlight recent outstanding DMM Resource articles that have the ultimate goal of benefitting of human health.

## Teaser

**Summary:** Disease Models & Mechanisms announces the launch of a new Outstanding Resource Paper Prize. To mark the occasion, we highlight exciting recent Resource articles and discuss their importance for biomedical research.

## Introduction

**Figure:**
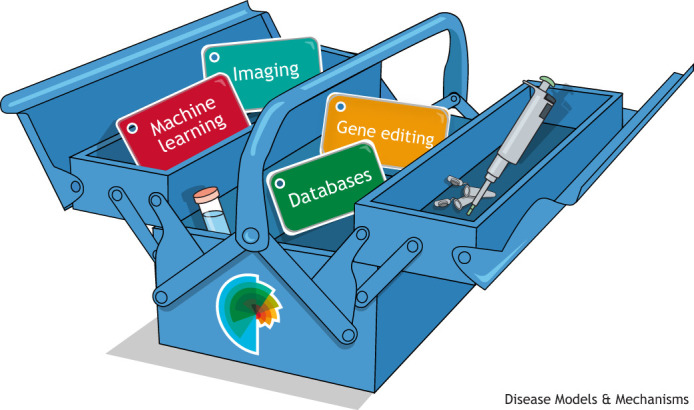


To achieve truly open science, the sharing of tools, reagents, datasets and methods is as important as the sharing of results. Accessible resources are, therefore, key to potentiating reproducible, reliable and rigorous disease biology research. Striving for integrity in science through openly accessible tools and resources can enable true advance in a given field and strengthen the translation of results to the clinic to benefit patients. Disease Models & Mechanisms (DMM)'s dedicated Resource articles report novel, or substantially improved, techniques, approaches, laboratory models, datasets (Cheng et al., 2022) or tools that are of significant use to a specific disease research community. In addition, rigorous validation of these advances is essential to a robust DMM Resource article to demonstrate its value in investigating disease mechanism and novel diagnostic or therapeutic approaches. Importantly, these articles and the data within are published fully Open Access, which maximises their impact on research, globally. In this Editorial, we summarise recent outstanding Resource articles published in DMM. Furthermore, in anticipation of future excellent Resource articles, DMM is launching a new ‘Outstanding Resource Paper Prize’ (Box 1).

**Box 1. DMM Outstanding Resource Paper Prize**

Each year, DMM awards a £1000 prize to the first author (or authors, where there is joint authorship) of the Research article that is judged by the journal's editors to be the most outstanding that year (Hackett, 2022, 2021). Starting with articles published in 2022, DMM will also award £1000 to the first author of the Resource article that is judged by the editors to be the most outstanding that year.

The shortlist of selected papers and first overall winner will be announced in DMM in 2023, along with a short profile of the winning author(s). Establishing a separate award for Resource articles allows us to showcase some of the exceptional Resource articles we publish that significantly contribute to the progression of disease research.

## Tracking, quantification and analysis of disease phenotypes

Biomedical tools and techniques continue to advance at a rapid pace and can greatly improve our ability to track disease phenotypes and responses to therapeutic intervention in diverse model systems. Specific examples include the evaluation of contrast-enhanced ultrasound as a non-invasive diagnostic tool in a rat model of hyperuricemic nephropathy (He et al., 2022), and the development of high-quality electrocardiogram and analysis methods for tracking cardiac abnormalities and testing novel drugs in adult zebrafish (Duong et al., 2021). Furthermore, Laird and colleagues developed a flow cytometry assay to assess protein aggregate formation in *in vitro* and *in vivo* models of spinocerebellar ataxia 3 (Robinson et al., 2021), and Resendiz-Sharpe et al. (2022) optimised a multimodal *in vivo* imaging strategy in a new mouse model of invasive pulmonary aspergillosis. Multi-omics are becoming a staple in comprehensive phenotyping studies; Houtkooper and colleagues have developed an analytical method to perform metabolomics and proteomics from a single *Caenorhabditis elegans* sample (Molenaars et al., 2021), increasing experimental efficiency and enriching the depth of information. Similar to *C. elegans*, yeast are a powerful tool for studying human disease (Kachroo et al., 2022). Loewen and team developed ‘sentinel’ yeast strains for functional assays of human disease genes that do not have endogenous yeast orthologues (Young et al., 2020).

Because of their genetic tractability and optical transparency, zebrafish provide unique insight into human disease. Reflecting the merits of this model system, DMM has published an array of Resource articles describing innovative gene-editing techniques to enable exploration of disease mechanisms in zebrafish. For example, Robles and colleagues developed a remarkable genetic labelling system for high-resolution *in vivo* imaging of dendritic spine development in zebrafish (DeMarco et al., 2022). The authors validated this technique in a zebrafish model of fragile X syndrome, but it has the potential to track dendritic spine abnormalities across many neurodevelopmental disorders and could enable high-throughput screening of novel treatments. Using fate-mapping techniques, Travnickova et al. (2022) tracked different cell populations in melanoma tumours with advanced imaging and flow cytometry to reveal new insights into tumour relapse after treatment. Similarly, the Lieschke group developed a flexible genome engineering approach to probe the function of individual immune cell populations in different physiological processes and disease states (Isiaku et al., 2021). The high-throughput capabilities of zebrafish tools have also allowed the development of an F0-based genetic assay that identified novel modifier genes of inherited cardiomyopathy (Ding et al., 2023).

From advanced imaging and flow cytometry techniques to exquisitely precise genetic manipulation, these Resource articles demonstrate that the toolkit available for researchers to interrogate human disease across diverse models of disease is continuing to expand.

## Expanding and enhancing the *in vivo* disease model repertoire

Advances in genome engineering allow researchers to improve the predictive validity of animal models (Hmeljak and Justice, 2019). For example, Nakahata et al. (2022) generated a genetically engineered mouse model of rhabdomyosarcoma, the most common soft tissue sarcoma in children, by introducing the K-Ras^G12D^ mutant and perturbing p53. This new mouse model recapitulated histological and transcriptomic features of human rhabdomyosarcoma, as well as responsiveness to targeted therapies. The temporal and spatial control of transgene expression can be achieved with Cre and CreER driver mouse lines, but this technique is not yet widely used when modelling neurodegeneration. Koller et al. (2022) employed this technique to manipulate amyloid precursor protein expression in a new mouse model of Alzheimer's disease that enables more precise investigation of amyloid pathology. Furthermore, Mito et al. (2022) demonstrated the use of sequential mutagenesis in adult zebrafish to model Spitz melanoma and reveal novel mechanisms in melanocytic lesion formation.

Other innovative approaches can also be employed to establish predictive disease models. The mitochondrial complex 1 inhibitor rotenone was used to replicate abnormal catecholamine metabolism in a novel rat model of Parkinson's disease (Landau et al., 2022). Also, surgical induction of blockages in blood flow to the left heart in foetal mice modelled hypoplastic left heart syndrome (Rahman et al., 2021). A particularly effective approach for generating clinically relevant animal models of cancer is the orthotopic xenografting of patient-derived tumour cells or biopsies. Ai et al. (2022) developed a robust protocol to xenograft patient-derived glioblastoma cells into zebrafish larvae brain and found that this model could recapitulate human glioblastoma and blood–brain barrier interactions to accurately predict long-term patient responses to chemotherapy, demonstrating its potential as a drug screening platform and tool for co-clinical trials. Similarly, Farre et al. (2021) generated a patient-derived orthotopic xenograft mouse model with a small extramedullary multiple myeloma biopsy that allowed both in-depth molecular analysis of the tumour and evaluation of drug responses. To improve the genetic diversity of xenograft models, Hasham and colleagues found that the genetic background of the host mice had a greater impact on phenotype than tumour type, with important implications for cancer modelling (Devlin and Roberts, 2022; Sargent et al., 2022). These techniques highlight how researchers can build a suite of complementary models that recapitulate heterogeneous patient populations.

Although novel animal models are advantageous for human disease research, several examples showcase how refining existing models can be an efficient approach to maximise our understanding of disease biology. The myotubularin knockout mouse has been widely used for neuromuscular disease studies, but there is a lack of understanding of the pathomechanisms or natural history of the disease. Sarikaya et al. (2022) performed a comprehensive longitudinal natural history of this X-linked myotubular myopathy model, informing the best functional, histological and molecular outcome measures for preclinical studies of this neuromuscular disorder.

Large animals can be excellent models of human disease, especially recapitulating neuromuscular disorders (van Putten, 2022). For example, a newly developed pig model of Duchenne muscular dystrophy accurately recapitulated not only the skeletal muscle pathology, but also the cardiac, behavioural and cognitive involvement, to potentiate large-scale and longitudinal studies (Stirm et al., 2021). Also, a new dog model of autosomal-dominant centronuclear myopathy recapitulated the functional and morphological muscle abnormalities of the human disease (Böhm et al., 2022). Using neuromuscular disorders as an example, it is clear that sharing data on thoroughly characterised animal models helps the field collectively progress to developing effective clinical approaches.

## Improved disease recapitulation with *in vitro* modelling

Advanced *in vitro* models, from cell lines and primary patient cells to organoids and bioengineered three-dimensional (3D) systems, offer invaluable tools to study molecular mechanisms of disease, cellular communication, and responses to microenvironmental stimuli and drugs. To better recapitulate the diversity of human disease, it is essential to expand genetic diversity in all model systems (Lek and Mardis, 2021), including *in vitro* disease modelling (Raghavan, 2022). Bönnemann and colleagues addressed this by using samples from genetically diverse patients with the rare neuromuscular disease α-dystroglycanopathy to generate a 3D *in vitro* system for the investigation of defects in the extracellular matrix and to evaluate a promising therapy (Nickolls et al., 2020). A similar drug-discovery platform that can advance cancer therapeutics is the MEMIC, a 3D-printed bioengineered *ex vivo* cellular system that allows researchers to interrogate the tumour microenvironment (Janská et al., 2021). To benefit traumatic brain injury research, the Finan group pioneered the first 3D human *in vitro* model of non-penetrating injury that reproduces several aspects of neurotrauma (Shoemaker et al., 2021). This offers a platform for studying the effects of blunt force injury on neurons and for developing therapies. Overall, *in vitro* systems have unique advantages, as they can model individual patient mutations, have expansive high-throughput capabilities and reduce our reliance on animal models. As bioengineering techniques continue to expand and improve, robust Resource articles with detailed protocols will be essential to enable reproducibility of these model systems.

## Computational resources to tackle expanding datasets

With the wealth of data generated by advanced technologies and high-throughput studies, Resource articles in DMM also describe new computational methods and resources to analyse these data. Dunne and colleagues have developed an intuitive and open-source data analysis platform for transcriptional data, called Molecular Subtyping Resource (Ahmaderaghi et al., 2022). This user-friendly platform enables in-depth analysis of RNA sequencing data from mouse and human tissue, and expands the accessibility of data analysis and interpretation. Another accessible analysis resource was developed by Murphy et al. (2022) and tackles inherent issues with statistical analysis of data generated from longitudinal studies. They developed and validated a linear mixed effects model that was better equipped to analyse correlated data from longitudinal studies compared to traditional two-way ANOVA. Resource articles can also utilise databases to provide new insights. Hansen et al. (2021) re-investigated an existing large-scale gene trap library of over 500,000 mouse embryonic stem cell lines and identified mutations in more than 2000 non-coding RNA genes; sharing their findings means that the corresponding cell lines can be used in *in vitro* and *in vivo* studies to interrogate the function of these genes in health and disease. Sharing user-friendly resources expands researchers' abilities to accurately analyse data and maximise their output, ensuring global accessibility.

## DMM's mission to promote accessible and reproducible science

The aims and scope of DMM are anchored in improving our understanding of human disease mechanisms, which is increasingly fuelling successful translation into the clinic (Justice et al., 2020). This effective translation relies on novel tools, models and resources with which to investigate human disease and test therapeutic interventions. Rendering these accessible to the broadest possible community of researchers will help ensure the validity of the results and robust translation. A reproducibility project found that only 46% of high-profile cancer biology papers assessed could be experimentally repeated, catalysing a movement to combat the reproducibility crisis in science (Rodgers and Collings, 2021). DMM has taken concrete steps in tackling this issue by mandating deposition of data and resources in a suitable repository. We have a long-lasting partnership with Dryad (https://journals.biologists.com/dmm/pages/journal-policies#data), which allows authors to deposit their data packages at the time of manuscript submission, making them securely available to referees (https://datadryad.org/stash). If and when the article is published in DMM, the data are automatically made public for mining and cross-referencing. Furthermore, all of our articles are published fully Open Access (Hackett and Patton, 2022), allowing researchers to freely share best practices in science and build upon knowledge. We invite authors to describe their novel resources and methods in sufficient detail to be easily replicated – and even refined for new applications – in other laboratories. Providing an adequate depth of detail is key to designing reproducible protocols, and to help authors achieve this, we have excluded the Materials and Methods section from the word count limits for all our Research and Resource papers.

DMM is proud to support the open science movement in the translational biomedical research space by publishing and promoting our Resource papers. The launch of our ‘Outstanding Resource Paper Prize’ will allow us to promote and encourage excellence in Resource articles (Box 1). This will support our goal to encourage collaboration, improve reproducibility and promote robust translation from bench to bedside.
